# Early discontinuation of endocrine therapy for breast cancer: who is at risk in clinical practice?

**DOI:** 10.1186/2193-1801-3-282

**Published:** 2014-06-04

**Authors:** Anna Kemp, David B Preen, Christobel Saunders, Frances Boyle, Max Bulsara, Eva Malacova, Elizabeth E Roughead

**Affiliations:** Centre for Health Services Research, School of Population Health, The University of Western Australia, 35 Stirling Hwy, Crawley, WA 6009 Australia; School of Surgery, The University of Western Australia, 35 Stirling Hwy, Crawley, WA 6009 Australia; Patricia Richie Centre for Cancer Care and Research, The Mater Hospital, University of Sydney, Rocklands Road, North Sydney, NSW 2060 Australia; Institute of Health and Rehabilitation Research, University of Notre Dame, PO Box 1225, Fremantle, WA 6959 Australia; Quality Use of Medicines and Pharmacy Research Centre, School of Pharmacy and Medical Sciences, University of South Australia, GPO Box 2471, Adelaide, SA 5001 Australia

**Keywords:** Persistence, Adherence, Tamoxifen, Aromatase inhibitors, 45 and up study

## Abstract

**Purpose:**

Despite evidence supporting at least five years of endocrine therapy for early breast cancer, many women discontinue therapy early. We investigated the impact of initial therapy type and specific comorbidities on discontinuation of endocrine therapy in clinical practice.

**Methods:**

We identified women in a population-based cohort with a diagnosis of early breast cancer and an incident dispensing of anastrozole, letrozole or tamoxifen from 2003–2008 (*N* = 1531). Pharmacy and health service data were used to determine therapy duration, treatment for pre-existing and post-initiation comorbidities (anxiety, depression, hot flashes, musculoskeletal pain, osteoporosis, vaginal atrophy), demographic and other clinical characteristics. Time to discontinuation of initial, and any, endocrine therapy was calculated. Cox regression determined the association of different characteristics on early discontinuation.

**Results:**

Initial endocrine therapy continued for a median of 2.2 years and any endocrine therapy for 4.8 years. Cumulative probability of discontinuing any therapy was 17% after one year and 58% by five years. Initial tamoxifen, pre-existing musculoskeletal pain and newly-treated anxiety predicted shorter initial therapy but not discontinuation of any therapy. Early discontinuation of any therapy was associated with newly-treated hot flashes (HR = 2.1, 95% CI = 1.3–3.3), not undergoing chemotherapy (HR = 1.4, 95% CI = 1.1–1.8) and not undergoing mastectomy (HR = 1.5, 95% CI = 1.2–1.8).

**Conclusions:**

Less than half of women completed five years of endocrine therapy. Women at greatest risk of stopping any therapy early were those with newly-treated hot flashes, no initial chemotherapy, or no initial mastectomy. This suboptimal use means that the reductions in recurrence demonstrated in clinical trials may not be realised in practice.

## Background

Endocrine therapy is the foundation of post-surgical treatment for women with hormone-dependent early breast cancer (Cheung 2007). Clinical trials clearly demonstrate that endocrine therapy halves the risk of recurrence and reduces cancer-related mortality when used for at least five years (ATAC Trialists’ Group 2005; Bliss et al. 2012; Coates et al. 2007; Davies and Adjuvant Tamoxifen: Longer Against Shorter (ATLAS) Collaborative Group 2013; Forbes et al. 2008; Gray and aTTom Collaborative Group 2013). Women using endocrine therapy for shorter time periods are at increased risk of recurrence and have higher mortality (Hsieh et al. 2014; McCowan et al. 2008; Swedish Breast Cancer Cooperative Group 1996; Yood et al. 2008). Despite strong empirical support for at least five years of endocrine therapy, early discontinuation of therapy in clinical practice has been reported in many European and North American studies (Hershman et al. 2010; Huiart et al. 2011; McCowan et al. 2008; Owusu et al. 2008; Partridge et al. 2008; van Herk-Sukel et al. 2010). A review of endocrine therapy duration of use outside of clinical trials reported that between 13% and 20% of women had discontinued therapy within one year, and 31%–73% had discontinued within five years (Murphy et al. 2012).

A number of demographic and clinical factors have been associated with early discontinuation including younger and older age (<45 years or >70 years) (Barron et al. 2007; Hershman et al. 2010; Nekhlyudov et al. 2011; van Herk-Sukel et al. 2010), being married (Kimmick et al. 2009), social disadvantage (Nekhlyudov et al. 2011), having a node-negative tumour (Kimmick et al. 2009), not undergoing chemotherapy or mastectomy (Barron et al. 2007; Hershman et al. 2010), and number of comorbidities (Demissie et al. 2001; Hershman et al. 2010; Kimmick et al. 2009; Neugut et al. 2011; van Herk-Sukel et al. 2010). Number of comorbidities has typically been ascertained using the Charlson comorbidity index (Charlson et al. 1987) or number of prescriptions used prior to commencing therapy, and reported associations with persistence to endocrine therapy have been mixed. Endocrine therapy may cause or exacerbate specific conditions including anxiety (Grunfeld et al. 2005; Güth et al. 2011), depression (Grunfeld et al. 2005), hot flashes (Grunfeld et al. 2005; Güth et al. 2011), musculoskeletal pain (Chim et al. 2013; Güth et al. 2011;), osteoporosis (Bell et al. 2013), and vaginal atrophy (Fallowfield et al. 2004). Self-report suggests that these comorbidities are the reason some women discontinue therapy (Murphy et al. 2012) but the role of specific comorbidities has not been examined in observational studies of discontinuation.

A number of observational studies of endocrine therapy duration have ended follow-up when a woman’s initial therapy is discontinued, reflecting the prominence of tamoxifen therapy until the mid-2000’s (Barron et al. 2007; McCowan et al. 2008; Partridge et al. 2003; van Herk-Sukel et al. 2010). There are few data to indicate whether characteristics associated with discontinuation of initial therapy (including initial therapy type) are associated with discontinuation of any endocrine therapy outside of clinical trials. One study of 4917 women with early breast cancer compared characteristics predicting discontinuation of initial therapy and any therapy (van Herk-Sukel et al. 2010). The authors reported that having ≥2 comorbidities was associated with shorter duration of any endocrine therapy but found no association with discontinuation of initial therapy (van Herk-Sukel et al. 2010). Another study compared duration of initial therapy in women commencing with tamoxifen or an aromatase inhibitor (AI: anastrozole, letrozole or exemestane) and reported greater discontinuation for tamoxifen users than those taking AIs at five years (31% vs. 19%) (Huiart et al. 2011).

The aim of this study was to determine duration of use for initial endocrine therapy, and any endocrine therapy by women treated for early breast cancer in clinical practice; and the impact of initial therapy type and specific comorbidities on early discontinuation.

## Methods

### Study population

Participants were drawn from the 45 and Up Study cohort, a population-based cohort of approximately 267,000 adults (143,014 women) aged ≥45 years residing in New South Wales (NSW), Australia (45 and Up Study Collaborators 2008). All cohort members provided written consent to join the 45 and Up Study, have their routinely-collected health data linked, and for these data to be provided to third-party researchers for approved projects.

### Data sources and linkage

We accessed unit-record, linked data from: i) the 45 and Up Study baseline survey, ii) NSW Central Cancer Registry, iii) NSW Admitted Patient Data Collection (all admissions to public and private hospitals), iv) Pharmaceutical Benefits Scheme (PBS) claims (all subsidised medicines), v) Medicare Benefits Schedule (MBS) claims (all subsidised outpatient consultations and investigations), and vi) NSW Registry of Births, Deaths, and Marriages. PBS and MBS data were supplied by the Department of Human Services, and remaining datasets were linked by the NSW Centre for Health Record Linkage (Centre for Health Record Linkage (CHeReL) 2009). The study period was defined as 1 January 2003 to 30 November 2011.

### Selection of cohort

The cohort comprised women in the 45 and Up Study with an incident dispensing of anastrozole, letrozole or tamoxifen between July 2003 and December 2008 and a diagnosis of invasive breast cancer listed on the Cancer Registry. A six month washout period from January-June 2003 was used to remove the ‘prevalent pool’ of women dispensed endocrine therapy. Women were excluded from the analysis if they had either a distant tumour at diagnosis, an initial dispensing date prior to diagnosis, or a period >18 months between diagnosis and commencing endocrine therapy. The final study sample comprised 1531 women.

### Determining duration of endocrine therapy use

The supply period for all medicines was considered to be pack size plus five days to allow for refill (i.e. 35 days) with the exception of tamoxifen 20 mg which was 65 days. Discontinuation was considered to have occurred if there was no dispensing of any endocrine therapy (anastrozole, exemestane, letrozole or tamoxifen) for a period of >180 days (Hershman et al. 2010; Nekhlyudov et al. 2011). Duration of endocrine therapy use was defined in two ways: i) time to last recorded dispensing of the participants’ initial endocrine therapy plus the supply period and ii) time to last recorded dispensing of any endocrine therapy plus the supply period.

### Ascertaining comorbidities, recurrence and death

Comorbid anxiety, depression, hot flashes, musculoskeletal pain, osteoporosis and vaginal atrophy were ascertained by claims for ≥1 specified PBS items dispensed during the study period (Table 1). PBS data do not capture information on indication, however, many items are restricted to use for particular indications. For example, PBS-supply of venlafaxine is restricted to use for major depressive disorders (Australian Government Department of Health 2013a). Comorbidities were considered to be ‘pre-existing’ if the relevant medication was dispensed before the incident endocrine therapy and ‘newly-treated’ if dispensed for the first time after endocrine therapy commenced. Breast cancer recurrence is not routinely collected in the Cancer Registry so was ascertained from: i) specified surgeries, chemotherapy or radiotherapy occurring for the first time >18 months from the date of diagnosis, or >12 months after previous claims for these events, or ii) first dispensing of a medicine indicated only for advanced breast cancer (Table 1). Fact and date of death were obtained from the death register for the years 2003–2010. Deaths occurring in 2011 were ascertained from PBS data by having no dispensing for any medicines for a period of >180 days. Deaths ascertained using this algorithm were validated against the death register for the period 2003–2010, with a resulting sensitivity and specificity of 98.2% and 90.2%, respectively. The algorithm detected 98% of deaths to within 1 calendar month of the date of death listed in the death register.

**Table 1:** **Medicine and service codes used to identify endocrine therapies, comorbidity and cancer recurrence, by data source**

Description	Name and item code
***Pharmaceutical benefits scheme***	
Endocrine therapies subsidised for early breast cancer	Anastrozole (8179L); exemestane (8506Q); letrozole (8245Y); tamoxifen (2109B, 2110C).
Anxiety treatments	Alprazolam (2130D, 2131E, 2132F, 8118G); diazepam (3161J, 3162K); oxazepam 3132W 3133X).
Depression treatments	Amitriptyline (2417F, 2418G, 2429W); citalopram (8220P, 8702B, 8703C); dothiepin (1357K, 1358L); doxepin (1011F, 1012G, 1013H); escitalopram (8700X, 8701Y, 9433L, 9711D, 9727Y, 9728B); fluoxetine (1434L, 8270G); fluvoxamine (8174F); imipramine (2421K); mianserin (1627P, 1628Q); moclobemide (1900B, 8003F); mirtazapine (8513C, 8855C, 8856D, 8857E, 8883M); nortriptyline (2522R, 2323T); paroxetine (2242B); reboxetine (8583R); sertraline (2236Q, 2237R, 8836C, 8837D); venlafaxine^a^ (8068P, 8301X, 8302Y, 8868R).
Hot flash treatments	Clonidine (3141H, 3145M).
Musculoskeletal pain treatments	Celecoxib (8439E, 8440F), diclofenac (1299J, 1300K, 1332D), ibuprofen (3190X, 3192B, 3198H, 5121M, 5124Q), indomethacin (2454E, 2757D); ketoprofen (1588N, 1590Q); meloxicam (8561N, 8562P, 8887R, 8888T); naproxen (1614Y, 1615B, 1659H, 1674D, 1795L); piroxicam (1895R, 1896T, 1897W, 1898X); rofecoxib (8471W, 8472X); sulindac (2047R, 2048T); tiaprofenic acid (2103Q).
Osteoporosis treatments	Alendronate (8102K, 8511Y, 9012H, 9183H, 9351E); etidronate (8056B); risedronate (8481J, 8621R, 8899J, 9147K, 9391G); strontium (3036T); zoledronate (9288W).
Vaginal atrophy treatment	Oestriol (1771F, 1776L, 1781R).
Medicines for advanced breast cancer^b^	Capecitabine (8362D, 8631C); lapatinib (9148L); medroxyprogesterone (2728N); megestrol (2734X), toremifene (8216K); vinorelbine (8280T, 8281W, 9009E, 9010F).
***Medicare benefits schedule***	
Chemotherapy	13915, 13918, 13921, 13924, 13927, 13930 13933, 13936.
Radiotherapy	15000-15012, 15100-15115, 15211-15217, 15219-15232, 15234-15247, 15249-15262, 15264-15272, 15303-15337, 15339-15357, 15339-15357.
***Admitted patient data collection***	
Services for cancer recurrence	Chemotherapy (13915, 13918, 13921, 13924, 13927, 13930 13933, 13936); lumpectomy (31500, 31503, 31506, 31509, 31512); mastectomy (31518, 31524); oophorectomy (35638, 35673, 35712, 35716, 35717, 35753, 35754).

^a^Venlafaxine subsidy is restricted to use for major depressive disorders but some off-label or dual use may occur for hot flashes.

^b^These therapies are not subsidised for the treatment of early breast cancer.

### Other covariates

Tumour stage and size were obtained from the Cancer Registry. Women undergoing mastectomy or chemotherapy prior to commencing endocrine therapy were identified through specified hospital and MBS items (Table 1). Specialty of the treating clinician was obtained from participant’s MBS claims between diagnosis and start of endocrine therapy (i.e. medical oncologist/other). Residential location was assessed using the Accessibility/Remoteness Index of Australia Plus (ARIA+) (Australian Population and Migration Research Centre 2013). Annual household income, highest level of education, marital status and country of birth were reported by participants at recruitment to the 45 and Up Study. County of birth was categorised as ‘Australia’, ‘New Zealand (NZ) or the United Kingdom (UK)’, or ‘other countries’.

### Statistical analysis

Kaplan-Meier curves were used to determine time from first endocrine therapy dispensing until discontinuation of initial therapy, and discontinuation of any therapy. Follow-up was censored at five years after initial endocrine dispensing, recurrence, death, or study end; whichever occurred first. Univariate and multivariate Cox-proportional hazards models were used to determine the association between a range of clinical and demographic characteristics with discontinuation of endocrine therapy before five years (early discontinuation). All variables were included in the multivariate (adjusted) models. Hazard ratios (HR) and 95% confidence intervals (CI) were calculated for each characteristic. All analyses were conducted using IBM SPSS version 19.

## Results

### Time to discontinuation of endocrine therapy

**Figure 1:**
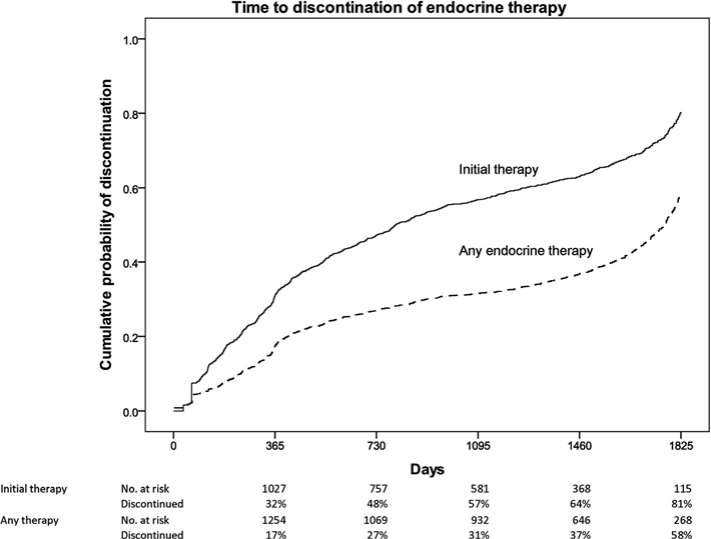
**Kaplan-Meier graph showing time to discontinuation of initial endocrine therapy, and any endocrine therapy.**

The median time for participants to discontinue their initial endocrine therapy was 2.2 years (range 0.8–4.9, Figure 1). Use of initial therapy had ceased for 32% of women at one year, increasing to 81% by five years. The median time for discontinuation of any endocrine therapy was considerably longer at 4.8 years. Cumulative probability of discontinuing any endocrine therapy was 17% after one year, 27% at two years, 31% at three years, and 58% by five years.

### Characteristics associated with discontinuation of initial therapy

Time to discontinuation of initial therapy varied by therapy type in both the unadjusted and adjusted models (Table 2). A history of anxiety, depression, hot flashes, osteoporosis or vaginal atrophy before commencing endocrine therapy did not impact on treatment duration. However, women with pre-existing musculoskeletal pain were significantly more likely to discontinue their initial therapy compared with other women (adjusted HR = 1.3, 95% CI = 1.1–1.5). Women newly-treated for anxiety, depression, flashes or vaginal atrophy after commencing endocrine therapy discontinued therapy earlier than women without these comorbidities but only newly-treated anxiety remained significant in the adjusted model (HR = 1.5, 95% CI = 1.1–1.9). Age, year of diagnosis, stage at diagnosis, no chemotherapy, no mastectomy and not seeing a medical oncologist were not related to duration of initial therapy in the adjusted model. Similarly, discontinuation of initial therapy was not associated with any demographic factors in the adjusted model including geographic remoteness, income, education, marital status or birth country.

**Table 2:** **Results of unadjusted and adjusted Cox proportional hazards models for discontinuation of initial or any endocrine therapy, by specified clinical and demographic characteristics**

Clinical and demographic characteristics	N (%)	Initial endocrine therapy		Any endocrine therapy	
		Unadjusted hazard ratio (95% CI)	Adjusted hazard ratio (95% CI)	Unadjusted hazard ratio (95% CI)	Adjusted hazard ratio (95% CI)
Initial therapy:					
Tamoxifen	917 (59.9%)	1.00	1.00	1.00	1.00
Anastrozole	518 (33.8%)	**0.53 (0.46–0.61)**	**0.57 (0.48–0.67)**	**0.64 (0.54–0.76)**	0.90 (0.74–1.09)
Letrozole	96 (6.3%)	**0.60 (0.45–0.80)**	**0.65 (0.49–0.87)**	0.75 (0.53–1.07)	1.04 (0.72–1.50)
Year of diagnosis:					
≥2004	1297 (84.7%)	1.00	1.00	1.00	1.00
<2004	234 (15.3%)	**1.77 (1.52–2.06)**	0.89 (0.63–1.25)	**2.26 (2.16–3.04)**	**1.56 (1.01–2.40)**
Age:					
<55 years	454 (29.7%)	1.00	1.00	1.00	1.00
55–74 years	878 (57.3%)	0.93 (0.82–1.07)	1.03 (0.88–1.20)	1.08 (0.91–1.28)	1.07 (0.88–1.29)
≥75 years	199 (13.0%)	**0.76 (0.62–0.94)**	0.79 (0.62–1.01)	1.22 (0.96–1.55)	1.31 (0.98–1.75)
Tumour size:					
>2 cm	533 (34.8%)	1.00	1.00	1.00	1.00
<1–2 cm	528 (34.5%)	1.02 (0.88–1.19)	0.94 (0.80–1.11)	1.09 (0.90–1.32)	0.90 (0.73–1.11)
≤1 cm	176 (11.5%)	1.01 (0.82–1.25)	0.84 (0.67–1.06)	**1.47 (1.15–1.90)**	1.13 (0.86–1.48)
Missing	294 (19.2%)	**1.75 (1.49–2.06)**	**1.43 (1.03–2.00)**	**2.52 (2.07–3.07)**	1.25 (0.82–1.91)
Stage:					
Node-positive	608 (39.7%)	1.00	1.00	1.00	1.00
Node-negative	865 (56.5%)	1.07 (0.94–1.21)	0.96 (0.84–1.10)	**1.37 (1.16–1.60)**	1.15 (0.97–1.36)
Missing	58 (3.8%)	1.16 (0.85–1.60)	0.89 (0.63–1.26)	1.28 (0.85–1.94)	0.73 (0.47–1.14)
No chemotherapy	1145 (74.7%)	**1.26 (1.09–1.46)**	1.05 (0.87–1.26)	**2.12 (1.72–2.62)**	**1.42 (1.11–1.83)**
No mastectomy	1103 (72.0%)	**1.21 (1.06–1.39)**	1.06 (0.91–1.23)	**1.77 (1.47–2.13)**	**1.45 (1.18–1.77)**
No medical oncologist^a^	922 (60.2%)	**1.16 (1.02–1.32)**	1.05 (0.90–1.23)	**1.84 (1.55–2.17)**	1.20 (0.98–1.48)
Pre-existing comorbidities:					
Anxiety	111 (7.3%)	1.00 (0.78–1.27)	1.00 (0.78–1.30)	0.99 (0.74–1.33)	1.09 (0.79–1.50)
Depression	271 (17.7%)	1.00 (0.85–1.18)	0.97 (0.81–1.16)	1.11 (0.91–1.35)	1.11 (0.91–1.36)
Hot flashes	14 (0.9%)	0.80 (0.40–1.60)	0.93 (0.46–1.90)	0.88 (0.39–1.96)	0.86 (0.38–1.95)
Musculoskeletal pain	368 (24.0%)	1.10 (0.96–1.27)	**1.25 (1.06–1.48)**	1.06 (0.89–1.27)	0.95 (0.77–1.17)
Osteoporosis	86 (5.6%)	0.81 (0.61–1.07)	0.77 (0.57–1.04)	0.91 (0.65–1.28)	0.84 (0.59–1.21)
Vaginal atrophy	43 (2.8%)	1.08 (0.74–1.57)	1.27 (0.87–1.87)	1.05 (0.65–1.67)	1.06 (0.66–1.72)
Newly-treated comorbidities:					
Anxiety	78 (5.1%)	**1.55 (1.20–1.99)**	**1.45 (1.11–1.90)**	**1.46 (1.08–1.97)**	1.09 (0.79–1.50)
Depression	271 (17.7%)	**1.19 (1.02–1.38)**	1.10 (0.93–1.30)	1.09 (0.90–1.31)	1.11 (0.91–1.37)
Hot flashes	28 (1.8%)	**1.56 (1.03–2.36)**	1.16 (0.76–1.79)	**2.33 (1.51–3.60)**	**2.07 (1.32–3.27)**
Musculoskeletal pain	328 (21.4%)	1.14 (0.99–1.32)	1.15 (0.98–1.35)	1.03 (0.86–1.23)	0.90 (0.73–1.09)
Osteoporosis	175 (11.4%)	0.89 (0.74–1.08)	0.93 (0.76–1.14)	0.94 (0.74–1.18)	0.88 (0.69–1.12)
Vaginal atrophy	60 (3.9%)	**1.56 (1.18–2.06)**	1.31 (0.98–1.75)	**1.56 (1.13–2.16)**	1.33 (0.95–1.88)
Location:					
Major city	690 (45.1%)	1.00	1.00	1.00	1.00
Regional	544 (35.5%)	1.05 (0.92–1.21)	1.08 (0.94–1.24)	1.12 (0.95–1.32)	1.10 (0.93–1.31)
Remote	297 (19.4%)	0.96 (0.81–1.13)	0.94 (0.79–1.12)	0.97 (0.79–1.19)	0.93 (0.75–1.15)
Household income:					
≥$70,000	272 (17.8%)	1.00	1.00	1.00	1.00
$30,000-$69,999	369 (24.1%)	0.95 (0.79–1.14)	0.94 (0.77–1.14)	1.04 (0.82–1.31)	1.02 (0.79–1.30)
<$30,000	477 (31.2%)	0.93 (0.78–1.11)	0.95 (0.76–1.17)	1.13 (0.91–1.41)	1.09 (0.83–1.42)
Refused	413 (27.0%)	**0.80 (0.67–0.96)**	0.82 (0.67–1.01)	0.96 (0.77–1.21)	0.90 (0.69–1.17)
Education:					
Undergraduate degree	330 (21.5%)	1.00	1.00	1.00	1.00
Certificate or diploma	395 (25.8%)	0.92 (0.77–1.09)	0.86 (0.72–1.03)	1.01 (0.82–1.25)	0.94 (0.75–1.17)
High school or less	777 (50.7%)	0.87 (0.75–1.02)	0.88 (0.74–1.04)	0.95 (0.79–1.15)	0.89 (0.72–1.10)
Missing	30 (2.0%)	0.84 (0.53–1.34)	0.85 (0.52–1.38)	1.03 (0.59–1.82)	0.90 (0.50–1.63)
Marital status:					
Partner or spouse	1084 (70.8%)	1.00	1.00	1.00	1.00
Single	79 (5.2%)	1.08 (0.83–1.41)	1.06 (0.80–1.40)	1.12 (0.81–1.55)	1.04 (0.74–1.46)
Separated or widowed	369 (24.1%)	0.93 (0.80–1.07)	0.95 (0.81–1.11)	1.00 (0.84–1.19)	0.87 (0.72–1.06)
Birth country:					
Australia	1143 (74.7%)	1.00	1.00	1.00	1.00
NZ, UK^b^	188 (12.3%)	1.14 (0.95–1.36)	1.19 (0.99–1.43)	**1.42 (1.14–1.76)**	**1.56 (1.16–2.10)**
Other	200 (13.1%)	0.94 (0.78–1.13)	0.90 (0.75–1.09)	0.93 (0.74–1.17)	1.04 (0.83–1.32)

(Bolded figures indicate *P* < 0.05).

^a^Prior to commencing endocrine therapy.

^b^New Zealand, United Kingdom.

### Characteristics associated with discontinuation of any endocrine therapy

Women commencing therapy with anastrozole were less likely to discontinue any endocrine therapy than those initiated on tamoxifen (unadjusted HR = 0.6, 95% CI = 0.5–0.8); however this relationship was not significant in the adjusted model (Table 2). None of the pre-existing comorbidities examined predicted duration of endocrine therapy in the unadjusted or adjusted models. Newly-treated anxiety, hot flashes and vaginal atrophy were associated with discontinuation of any therapy in the unadjusted models but only hot flashes remained significant after adjusting for other variables (HR = 2.1, 95% CI = 1.3–3.3).

Clinical characteristics including age, tumour size and stage were not associated with discontinuation of therapy in the adjusted models. However, women not undergoing chemotherapy or mastectomy prior to endocrine therapy were significantly more likely to discontinue therapy by five years compared with women who did receive these treatments (adjusted model, chemotherapy HR = 1.4, 95% CI = 1.1–1.8; mastectomy HR = 1.5, 95% CI = 1.2–1.8). Women born in the UK or NZ were significantly more likely to discontinue endocrine therapy than Australian-born women (HR = 1.6, 95% CI = 1.2–2.1). No other demographic characteristics were related to duration of therapy in the unadjusted or adjusted models.

## Discussion

Only 19% of women receiving endocrine therapy for early breast cancer in clinical practice continued to use their initial therapy for five years. When allowing for switching between therapies, the proportion of women using any endocrine therapy for five years, as consistently recommended by clinical trial evidence (ATAC Trialists’ Group 2005; Bliss et al. 2012; Coates et al. 2007; Davies and (ATLAS) (2013); Forbes et al. 2008; Gray and aTTom Collaborative Group 2013), was 42%. These suboptimal persistence rates are similar to discontinuation rates of 31–73% by five years reported elsewhere (Murphy et al. 2012). The extent of early discontinuation of all endocrine therapy observed here suggests that more than half of treated women do not obtain the benefits of endocrine therapy demonstrated in clinical trials (Hsieh et al. 2014; McCowan et al. 2008; Swedish Breast Cancer Cooperative Group 1996; Yood et al. 2008). Given that tamoxifen duration is now recommended to be 10 years based on ATLAS and aTTom data, this suboptimal efficacy is likely to be magnified in the future (Davies and (ATLAS) (2013); Gray and aTTom Collaborative Group 2013).

We observed different predictors for discontinuation of initial therapy compared with any endocrine therapy. Women were more likely to discontinue their initial therapy if they commenced with tamoxifen, had pre-existing arthritis, or were newly-treated for anxiety. The finding that women discontinued initial therapy with tamoxifen earlier than women initiated on anastrozole or letrozole is unsurprising given that trial evidence and clinical guidelines support a switch from tamoxifen to an aromatase inhibitor after 2–3 years for post-menopausal women (Baum et al. 2003; National Breast Cancer Centre (NBCC) and National Health and Medical Research Council (NHMRC) 2001; Winer et al. 2005). Given that tamoxifen is the only endocrine therapy recommended for pre- or peri-menopausal, it is more likely that women would switch from tamoxifen to AIs as therapy progresses than the converse (Cheung 2007; NBCC and NHMRC 2001). Switching from tamoxifen to an AI, rather than the converse, is also more likely because our study covers the period before release of the BIG 1–98 results, which supports use of AIs before a switch to tamoxifen in low and intermediate risk women (Viale et al. 2011). It is encouraging to note that initial therapy type did not influence the total duration of endocrine therapy for women in this study, nor did the other predictors of initial therapy discontinuation: pre-existing musculoskeletal pain and newly-treated anxiety.

Our results showed that the women most likely to discontinue any endocrine therapy before five years were those who did not undergo chemotherapy or mastectomy. This finding remained after adjusting for treatment by a medical oncologist. Previous studies have reported that women not undergoing chemotherapy or mastectomy are more likely to discontinue therapy early. A large observational study of 8769 women followed for 4.5 years found that women undergoing chemotherapy before commencing endocrine therapy were significantly less likely to discontinue than other women, and those who had undergone mastectomy were less likely to discontinue than those who had a lumpectomy (Hershman et al. 2010). A shorter-term study (1 year follow-up) of >13,000 women also reported that women with pre-endocrine therapy mastectomy had nearly half the probability of non-adherence compared with women without mastectomy (Sedjo and Devine 2011). It may be that women who undergo more ‘aggressive’ treatments perceive their condition as more serious and their need for endocrine therapy greater, than other women.

Women in this study first treated for hot flashes after commencing endocrine therapy were at higher risk of early discontinuation than other women. Hot flashes were the side-effect women most commonly cited for discontinuing tamoxifen in Swiss (*N* = 400) and UK studies (*N* = 110) (Charlson et al. 1987; Neugut et al. 2011). Anecdotally, women describe hot flashes as causing sleep disturbance, difficulty concentrating and decreased quality of life. It is interesting to note that only newly-treated, and not pre-existing, hot flashes were associated with early discontinuation. None of the other pre-existing or newly-treated comorbidites we examined were associated with early discontinuation of any endocrine therapy. We may have underestimated treatment for hot flashes in this group because our data did not include non-PBS supply of medicines which might be used for flushing (such as gabapentin and venlafaxine, which are restricted to use for epilepsy and major depression, respectively) (Australian Government Department of Health 2013a).

It is not clear why women born in NZ or the UK would discontinue endocrine therapy earlier than Australian-born women. All of the women in the study were residents of NSW at recruitment to the 45 and Up Study (2006–2009) and reported a median duration of residence in Australia of 37 years (range 26–47). NZ and the UK are culturally similar to Australia, have comparable public pharmaceutical insurance (Kemp et al. 2010), and NZ and UK immigrants to Australia are the most frequent of any countries (Australian Bureau of Statistics 2013). Therefore we consider it unlikely that social marginalisation or unfamiliarity with the health system underpins this higher discontinuation. Further research with this group of women is needed to understand the increased risk of early discontinuation observed here or indeed see if this represents just a spurious finding.

### Strengths and weaknesses

To our knowledge, this is the first observational study with follow-up >2 years to examine the impact of initial therapy and specific comorbidities on discontinuation of initial endocrine therapy and any endocrine therapy. Many studies with long follow-up have only examined tamoxifen discontinuation (Barron et al. 2007; Huiart et al. 2011; McCowan et al. 2008; Partridge et al. 2003), and have examined number of comorbidities rather than specific conditions (Kimmick et al. 2009; McCowan et al. 2008; Nekhlyudov et al. 2011; Neugut et al. 2011; van Herk-Sukel et al. 2010; Weaver et al. 2013). Our study is also the first observational study investigating duration of endocrine therapy in Australian clinical practice. Australia’s PBS provides subsidised prescription medicines for all Australian residents at relatively low cost (up to AUS^a^ $5.60 and $34.20 per item for social security recipients and other beneficiaries, respectively, as of study end in 2011 (Australian Government Department of Health 2013b; Organisation for Economic Co-operation and Development 2014); therefore cost may be less of a barrier to use in Australia than in some other countries (Kemp et al. 2011).

We used health records for a heterogeneous community sample for whom all publically-subsidised endocrine therapies have been captured. We were able to follow women for up to five years after commencing their initial therapy, with a minimum follow-up time of 2.9 years. The findings reported here are comparable to those in previous observational studies of discontinuation as the same definition of discontinuation has been used (180 days), and follow-up was similarly censored for recurrence and death (Barron et al. 2007; Hershman et al. 2010; Nekhlyudov et al. 2011; van Herk-Sukel et al. 2010). Women experiencing troubling side-effects may take a ‘drug holiday’ from endocrine therapy for up to 3 months (Jordan et al. 2011; Sehdev et al. 2009), and so our use of 180 days to define discontinuation would avoid misclassification of these women.

There are limitations to this study. Our sample was drawn from the 45 and Up Study, limiting the sample to individual’s ≥45 years and consenting to linkage of their health records. The health service history of such individuals may differ from younger people, or those who do not agree to participate in cohort studies. Although we could not directly ascertain whether participants had hormone-dependent tumours, endocrine therapies in Australia are only publically-subsidised for hormone-dependent tumours (Australian Government Department of Health 2013a). We could not directly assess cancer recurrence and had to ‘triangulate’ using medicines and services used to treat recurrence. This method of ascertaining breast cancer recurrence has been successfully demonstrated using administrative health service data in previous studies (Earle et al. 2002; Lamont et al. 2006; Stokes et al. 2008). The validity of recurrence using this method has also been compared with chart review with a reported sensitivity and specificity of 92% and 94% respectively (Earle et al. 2002).

We were limited to analysing dispensing of endocrine therapy rather than actual consumption. It is not possible to determine whether prescriptions were filled but not taken by women in this study, and similarly women who were prescribed but never dispensed their initial endocrine therapy were not captured. Despite this limitation, prescription refills are used as a measure of adherence and persistence in many endocrine therapy duration studies (Hershman et al. 2010; Huiart et al. 2011; Murphy et al. 2012; van Herk-Sukel et al. 2010) and are consisted an objective and accurate proxy for actual intake (NBCC and NHMRC 2001).

## Conclusion

Less than half of women in this study (42%) completed five years of endocrine therapy for breast cancer. This suboptimal use means that the full benefit of endocrine therapies in reducing recurrence and cancer-related mortality consistently demonstrated in clinical trials may not be realised in practice. Choice of initial endocrine therapy, pre-existing musculoskeletal pain and newly-treated anxiety were related to shorter duration of initial therapy but not early discontinuation of any therapy. Women at greatest risk of stopping any endocrine therapy before the recommended five years were those receiving less aggressive initial treatment (i.e. no chemotherapy or mastectomy), those with newly-treated flashes, and those born in UK or NZ. Strategies are needed to support medicine use and improve outcomes for these women.

### Ethics statement

This study was conducted in accordance with Australian law. The consent procedure for entry to the 45 and Up Study was approved by the University of NSW Human Research Ethics Committee and the Australian Government Department of Health and Ageing. The current study also received approval from The University of Western Australia Human Research Ethics Committee (approval RA/4/1/4589), and the NSW Population and Health Services Research Ethics Committee (approval HREC/11/CIPHS/35).

## Endnote

^a^In 2011, the purchasing power of 1$US was 1.51 AUS dollars.
